# The voluntary HIV counselling and testing service in Kenema District, Sierra Leone, 2004-2006: a descriptive study

**DOI:** 10.1186/1472-698X-10-4

**Published:** 2010-03-09

**Authors:** Fiona G Kouyoumdjian, Alhassan L Seisay, Brima Kargbo, Sheik H Khan

**Affiliations:** 1 Department of Epidemiology, Dalla Lana School of Public Health, University of Toronto, 155 College Street, Toronto, M5T 3M7, Canada; 2 Disease Prevention and Control, Ministry of Health & Sanitation, Freetown, Sierra Leone; 3 National HIV/AIDS Secretariat, Freetown, Sierra Leone; 4 HIV/AIDS Treatment and Lassa Fever Programme, Kenema Government Hospital, Kenema, Sierra Leone

## Abstract

**Background:**

Voluntary counselling and testing (VCT) is an important component of national HIV programs, which are necessary to realize the right to health. VCT data also provide valuable information on regional HIV epidemiology.

**Methods:**

The study examines data on the population that obtained HIV VCT in Kenema District, Sierra Leone, from 2004 to 2006, using descriptive statistics and exploring potential HIV risk factors using bivariate and multivariable logistic regression. Analysis was performed separately for two subpopulations: those accessing VCT routinely as part of antenatal care and those specifically seeking VCT.

**Results:**

During this period, 2230 people accessed VCT: 1213 through antenatal testing and 1017 specifically seeking VCT. The HIV prevalence was 0.6% in women presenting for antenatal care, 12.6% in women specifically accessing VCT, and 6.7% in men specifically accessing VCT. In both bivariate and multivariable analyses, being female was statistically significantly associated with testing positive in people specifically seeking VCT.

**Conclusions:**

These data from the VCT service in Kenema will be used to improve the accessibility of HIV testing. Questions raised by the analysis will be used to enhance data collection and to inform further research on risk factors.

## Background

Sierra Leone is a country in West Africa with a population of 5.9 million, 2.7 million of whom are aged 15-49 [1]. Subsequent to a civil war that ended in 2001, health indicators for Sierra Leone reveal poor average health, with a life expectancy at birth of 40 years, an infant mortality rate of 159 per 1000 live births, an under-5 mortality rate of 269 per 1000 live births, and a maternal mortality ratio of 2100 per 100,000 [1].

While the HIV/AIDS epidemic is profoundly affecting many parts of sub-Saharan Africa including countries in West Africa, relatively little is known about the epidemiology of HIV/AIDS in Sierra Leone. A national population-based seroprevalence study in 2005 revealed a prevalence of 1.53% [2], with no significant difference in prevalence between women and men. Of those who tested positive for HIV, 91% were HIV-1 positive, 4.5% were HIV-2 positive, and 4.5% were positive for both HIV-1 and HIV-2. The World Health Organization's 2008 update for Sierra Leone indicates that the infection rate for adults aged 15 to 49 was between 1.3% and 2.4% in 2007, as compared with a rate between 0.7% and 2.1% in 2001, with an overall increase in prevalence since 1990 [1]. The update also states that in 2006, the HIV prevalence for pregnant women at sentinel surveillance sites was between 4.4% and 5.4% in urban areas and between 1.7% and 8.9% outside major urban areas. Further research is clearly needed to define the overall burden of disease and to identify risk factors with greater precision.

In the context of an apparently worsening HIV/AIDS epidemic in Sierra Leone, it is important to recognize that health is an inalienable human right, as enshrined in the Universal Declaration of Human Rights (UDHR) and the International Covenant on Economic, Social, and Cultural Rights (ICESCR). Article 25 of the UDHR states that people have the right "to a standard of living adequate for the health and well-being of [themselves and their families], including... medical care and necessary social services." [3] Article 12 of the ICESCR articulates the right to "enjoyment of the highest attainable standard of physical and mental health," and specifies that States Parties to the Covenant shall take steps to prevent, treat, and control epidemic diseases, and to create conditions to "assure to all medical service and medical attention in the event of sickness." [4] Sierra Leone ratified the ICESCR in 1996, and has much work to do to progressively realize the goal of the right to health for all.

In order to respect, protect, and fulfill the right to health, States such as Sierra Leone need to define their health status and develop strategies to improve morbidity and mortality, including with respect to HIV/AIDS. One important component of HIV/AIDS prevention and care services is voluntary counselling and testing (VCT). VCT has been shown to reduce HIV risk in various countries [5,6] and is widely recommended as a prevention strategy, although evidence on the effectiveness of VCT as a risk prevention intervention is inconsistent [7-9]. VCT may also contribute to primary, secondary, and tertiary prevention of HIV/AIDS by decreasing stigma, encouraging community support and care, and serving as an entry point to access social and medical services for people affected by HIV [10]. Of note, in Sierra Leone, antiretroviral therapy is provided at no cost to eligible patients, therefore access to diagnosis through VCT programs also frequently means access to antiretroviral treatment when indicated.

States also need to define their health status in order to identify opportunities for intervention and to measure the effectiveness of policies and programs. Data gathered by VCT services provide useful information about who is accessing VCT, about HIV prevalence, and about potential HIV risk factors. These data can be used to assess and enhance the VCT service itself, as well as to inform the development of community education, outreach, service initiatives, and further research.

With this context in mind, the objective of this study was to explore data gathered from 2004 to 2006 by the VCT service in Kenema District, Sierra Leone. Specifically, we describe the demographic characteristics of those seeking testing, calculate the prevalence of HIV, and explore potential risk factors for HIV.

## Methods

### Setting

Kenema District has an estimated population of 350,000 [11] as of 2007 and an HIV prevalence of 1.9% as per the 2005 national survey [2]. The Kenema Government Hospital HIV VCT program was developed in March 2004 and is the only HIV testing site in Kenema District. The VCT program routinely collects demographic information about its clients, including gender, age, religion, marital status, and occupation, and these data are linked with HIV test results.

### Study Population

All people who accessed VCT at Kenema Government Hospital from the development of the VCT program in March 2004 until March 2006 were included in the study. There are two groups of people who obtain VCT: women accessing antenatal care at Kenema Government Hospital, who are offered opt-in testing as routine care, and people who specifically seek VCT at the hospital.

### Data Analysis

Analysis was conducted separately for people who accessed VCT as part of routine antenatal care and for those who accessed VCT outside of this program. For those who specifically accessed VCT, the descriptive analysis was stratified by gender. Age was reported as a continuous variable but was categorized for the analysis. More than 80 types of work were reported by clients. Since there was no clear way to classify types of work, only the 5 occupations which were reported by more than 100 persons were assessed independently, and the remaining occupations were combined into a single category of "other."

Statistical analysis was performed using Stata version 10, and included calculating frequencies of various demographic factors and the prevalence of HIV in the population; chi-squared tests to compare proportions; and bivariate and multivariable logistic regression to explore risk factors for testing positive for HIV.

Since the goal of risk factor analysis was to explore potential risk factors and not to confirm a specific hypothesis, in the multivariable analysis, all variables were added simultaneously into a model. To examine for potential confounding between the variables in the model, variables were removed from the model individually and then in groups to look for changes to the beta of greater than 10% of any of the remaining variables. A final model was then run which contained only variables which were statistically significantly associated with testing positive for HIV or which functioned as confounders of variables in the model.

The study was approved by the Research and Ethics Committee of the Sierra Leone Ministry of Health and Sanitation.

## Results

From 2004 to 2006, 2230 people obtained VCT, 1213 through the antenatal testing program and 1017 specifically seeking VCT.

### Antenatal population accessing VCT

Of the 1213 women who obtained VCT as part of routine antenatal care, 27.5% were 15 to 19, 31.4% were 20 to 24, 23.2% were 25 to 29, and 10.3% were 30 to 34 years old, as shown in Table 1. Almost 80% of the population was Muslim, and 16.6% was Christian. The vast majority of these women were married, at 77.7%, and of those who were married, 72.2% were in monogamous marriages and 27.7% were in polygamous marriages. In terms of occupation, 36.4% were housewives, 20.3% were traders, and 12.5% were farmers.

**Table 1:** Antenatal population: Characteristics and unadjusted and adjusted OR for HIV

Variable		Number (%)	HIV+ n (%)	Unadjusted OR (95%CI)	Adjusted OR (95%CI)
Total		1213 (100)	7 (0.6)	-	-

Age Category (years)	<15	1 (0.1)	0 (0)	-	-
	
	15-19	334 (27.5)	0 (0)	-	-
	
	20-24	381 (31.4)	4 (1.0)	1	1
	
	25-29	282 (23.2)	2 (0.7)	0.7 (0.1, 3.7)	0.6 (0.1, 3.7)
	
	30-34	125 (10.3)	1 (0.8)	0.8 (0.8, 6.9)	0.7 (0.1, 7.1)
	
	35-39	70 (5.8)	0 (0)	-	-
	
	40-44	13 (1.1)	0 (0)	-	-
	
	45-49	1 (0.1)	0 (0)	-	-
	
	>49	1 (0.1)	0 (0)	-	-

Religion	Muslim	946 (78)	5 (0.5)	1	1
	
	Christian	201 (16.6)	2 (1)	1.9 (0.4, 9.8)	2.1 (0.4, 12.7)

Marital Status	Married monogamous	680 (56.1)	3 (0.4)	1	1
	
	Married polygamous	261 (21.5)	3 (1.1)	2.6 (0.5, 13.2)	2.6 (0.5, 13.8)
	
	Single	186 (15.3)	1 (0.5)	1.2 (0.1, 11.8)	1.8 (0.1, 62.5)
	
	Divorced	0 (0)	0 (0)	-	-
	
	Widowed	0 (0)	0 (0)	-	-
	
	Separated	1 (0.1)	0 (0)	-	-

Occupation	Student	151 (12.5)	1 (0.7)	1	1
	
	Farmer	103 (8.5)	1 (1)	1.5 (0.1, 24.0)	1 (0.0, 53.3)
	
	Businessperson	78 (6.4)	0	-	-
	
	Trader	246 (20.3)	0	-	-
	
	Housewife	441 (36.4)	4 (0.9)	1.4 (0.2, 12.4)	1.1 (0.0, 38.8)
	
	Other	122 (10.1)	1 (0.8)	1.2 (0.1, 20.0)	0.7 (0.0, 25.6)

The HIV prevalence was 0.6% in women presenting for antenatal care. Of the 7 women who tested positive, 4 tested positive for HIV-1 and 3 tested positive for both HIV-1 and HIV-2. In exploratory regression analysis, none of the demographic characteristics was found to be a statistically significant risk factor for HIV infection in either bivariate or multivariable analysis, as shown in Table 1.

### Non-antenatal population accessing VCT

The characteristics of the males and females specifically seeking VCT are shown in Table 2. More than half of the population was female. For the 533 females, more than two thirds were Muslim. There was a broad distribution of females across age categories from ages 15 and above, however there were only 12 people tested who were younger than 15. Forty-four point one percent of the females were in monogamous marriages, 24.4% were in polygamous marriages, and 20.8% were single. Regarding occupation, 25.7% of females were farmers, 19.7% were housewives, 14.6% were traders, and 20.5% had other occupations.

**Table 2:** Non-antenatal population: Characteristics and HIV prevalence, by sex

Variable		Female		Male	
		
		Number (%)	HIV+ n (%)	Number (%)	HIV+ n(%)
Total		533 (52.4)	67 (12.6)	484 (47.6)	32 (6.7)

Age Category (years)	<15	12 (2.3)	1 (8.3)	14(2.9)	3 (23.1)
	
	15-19	49 (9.2)	2 (4.1)	43(8.9)	1 (2.3)
	
	20-24	78 (14.6)	10 (13)	53(11)	2 (3.9)
	
	25-29	106 (19.9)	11 (10.4)	72(14.9)	4 (5.6)
	
	30-34	70 (13.1)	12 (17.1)	68(14.1)	4 (5.9)
	
	35-39	50 (9.4)	9 (18)	74(15.3)	4 (5.4)
	
	40-44	43 (8.1)	7 (16.3)	42(8.7)	3 (7.5)
	
	45-49	40 (7.5)	4 (10)	31(6.4)	2 (6.5)
	
	>49	56 (10.5)	7 (12.5)	67(13.8)	8 (11.9)

Religion	Muslim	378 (70.9)	55 (14.6)	330(68.2)	21 (6.4)
	
	Christian	87 (16.3)	11 (12.8)	90(18.6)	8 (9)

Marital	Married monogamous	235 (44.1)	28 (11.9)	231(47.7)	13 (5.7)
	
	Married polygamous	130 (24.4)	12 (9.2)	41(8.5)	1 (2.4)
	
	Single	111 (20.8)	13 (11.8)	175(36.2)	10 (5.8)
	
	Divorced	10 (1.9)	2 (20)	14(2.9)	2 (14.3)
	
	Widowed	26 (4.9)	7 (26.9)	4(0.8)	1 (25)
	
	Separated	3 (0.6)	2 (66.7)	1(0.2)	0

Occupation	Housewife	105 (19.7)	8 (7.6)	0	0
	
	Trader	78 (14.6)	99 (11.7)	42(8.7)	3 (7.3)
	
	Student	57 (10.7)	5 (8.8)	80(16.5)	4 (5.1)
	
	Farmer	137 (25.7)	16 (11.8)	90(18.6)	4 (4.4)
	
	Businessperson	24 (4.5)	7 (29.2)	19(3.9)	2 (10.5)
	
	Other	109 (20.5)	15 (13.8)	229(47.3)	1 (7.5)

For the 484 males, the distribution by religion was similar to the distribution for females, with 68.2% of the population reporting being Muslim and 18.6% of the population reporting being Christian. Also similar to females, there was a broad distribution across age categories, with the notable exception that only 14 males younger than 15 were tested. Almost half of the population was in a monogamous marriage, 8.5% reported being in a polygamous marriage, and 36.2% were single. Almost half of the men had other occupations, but 18.6% were farmers, 16.5% were students, and 8.7% were traders.

The overall prevalence of HIV was 9.7% in the population specifically seeking VCT, which is significantly higher than the national prevalence in the 2005 national population-based survey, which was 1.53% (χ^2^(1df) = 263.3, p < 0.01). As shown in Table 2, 12.6% of women and 6.7% of men tested positive. Of the 99 people who tested positive, 52.5% were positive for HIV-1, 18.2% were positive for HIV-2, and 29.3% were positive for both HIV-1 and HIV-2.

In exploratory regression analysis, being female (OR 2.0, 95% CI 1.3, 3.1); being 15-19 years old compared to being younger than 15 (OR 0.2, 95% CI 0.0, 0.9); being widowed (OR 3.8, 95% CI 1.6, 9) or separated (OR 10.3, 95% CI 1.4, 75.2) compared to being in a monogamous marriage; and being a businessperson (OR 3.7, 95% CI 1.4, 10.1) were significantly associated with testing positive in bivariate analysis, as shown in Table 3.

**Table 3:** Non-antenatal population: Unadjusted and Adjusted ORs of Testing Positive

Variable		Unadjusted OR (95%CI)	Adjusted OR (95%CI)	Final Model
Gender	Male			
	
	Female	2.0 (1.3, 3.1)**	2.4 (1.4, 3.9)**	2.3 (1.4, 3.6)**

Age Category (years)	<15	1	1	1
	
	15-19	0.2 (0.0, 0.9)*	0.3 (0.0, 1.4)	0.3 (0.1, 1.4)
	
	20-24	0.5 (0.2, 1.8)	0.8 (0.2, 2.9)	0.8 (0.2, 3.1)
	
	25-29	0.5 (0.16, 1.6)	0.7 (0.2, 2.7)	0.8 (0.2, 3.1)
	
	30-34	0.7 (0.2, 2.3)	1.2 (0.3, 4.8)	1.4 (0.4, 5.2)
	
	35-39	0.6 (0.2, 2.1)	1.1 (0.3, 4.5)	1.3 (0.3, 5.0)
	
	40-44	0.7 (0.2, 2.5)	1.1 (0.3, 4.9)	1.3 (0.3, 5.5)
	
	45-49	0.5 (0.1, 1.9)	0.6 (0.1, 2.8)	0.8 (0.2, 3.5)
	
	>49	0.7 (0.2, 2.4)	1.1 (0.3, 4.5)	1.2 (0.3, 4.8)

Religion	Muslim	1	1	-
	
	Christian	1.0 (0.6, 1.7)	1.0 (0.6, 1.8)	-

Marital Status	Married monogamous	1	1	1
	
	Married polygamous	0.8 (0.4, 1.6)	0.6 (0.3, 1.3)	0.7 (0.3, 1.3)
	
	Single	0.9 (0.5, 1.6)	1.4 (0.7, 2.8)	1.4 (0.8, 2.6)
	
	Divorced	2.1 (0.7, 6.3)	1.7 (0.5, 5.5)	2.1 (0.7, 6.5)
	
	Widowed	3.8 (1.6, 9)**	2.4 (0.9, 6.5)	2.8 (1.1, 7.1)
	
	Separated	10.3 (1.4, 75.2)*	8.2 (1.0, 63.4)*	10.4 (1.4, 77.7)*

Occupation	Student	1	1	-
	
	Farmer	1.4 (0.6, 3.1)	1.0 (0.4, 2.9)	-
	
	Businessperson	3.7 (1.4, 10.1)*	2.3 (0.7, 7.7)	-
	
	Trader	1.6 (0.6, 3.9)	1.2 (0.4, 3.5)	-
	
	Housewife	1.2 (0.4, 3.1)	0.8 (0.2, 2.5)	-
	
	Other	1.5 (0.7, 3.2)	2.1 (0.6, 7.0)	-

*p < 0.05, **p < 0.01

In a multivariable analysis, only being female (OR 2.4, 95% CI 1.4, 3.9) and being separated (OR 8.2, 95% CI 1.0, 63.4) were statistically significantly associated with testing positive. Age category was found to confound the relationship between sex and HIV status, and a final model including only age category, marital status, and sex resulted in an OR of 2.3 for females (95% CI 1.4, 3.6) compared to males and of 10.4 for people who were separated (95% CI 1.4, 77.7) compared to those in monogamous marriages, as shown in Table 3.

## Discussion

This study reveals valuable information and raises important questions for the VCT program at Kenema District Hospital, as well as for regional HIV efforts.

To realize the right to health, States need to identify groups at risk of HIV in order to develop effective HIV prevention and control programs. In comparison with the 2005 population-based estimates for Sierra Leone and for Kenema District, this study found a high prevalence of HIV in women and men who specifically sought VCT, though not in women accessing VCT antenatally. This relatively high prevalence may be due to the fact that people often seek VCT because of known risk factors [12], which would enrich the population seeking VCT for people more likely to be positive. The increased prevalence could putatively also be due to a relatively high rate of HIV in the general population in Kenema or in subpopulations within Kenema. Further support for this possibility of regional variation in the epidemiology of HIV is that there was a high proportion of HIV-2 in the overall population accessing VCT in Kenema, with 47.2% of infected persons testing positive for either HIV-2 alone or HIV-1 and HIV-2, compared with only 9% in the 2005 national sample [2]. These findings demand further investigation in order to ensure the implementation of effective prevention programs and services, including treatment, throughout Sierra Leone.

As noted already, VCT services may provide a means for States to fulfill the right to health as a way to prevent HIV and as the gateway for people with HIV to access health care. However, States must ensure that VCT is accessible and acceptable to the whole population at risk of HIV. This study reveals that relatively few men and children accessed VCT during the study period. While women of reproductive age have facilitated access to VCT as a routine part of antenatal care, there is no such linkage of VCT to routine services for men. Men may face barriers to VCT such as stigma and a lack of knowledge. It is also concerning that so few children were tested during the study period, especially given the number of women who tested positive antenatally and otherwise, since post-test counselling should recommend that women test their children when indicated. The Kenema VCT program should reach out to populations who may experience stigma or may lack knowledge about testing, should work to define and improve the accessibility and acceptability of the service, and should discuss the need for testing children and sexual partners in pre- and post-test counselling when indicated.

Notably, the study is limited by the lack of detail in the routinely collected data. Specifically, it was not possible to determine whether there were people who sought testing more than once within the study period, which could affect the estimates generated in this study. Further, no data were collected about why people sought HIV testing and about risk behaviours, which would be helpful in contextualizing why people are seeking VCT and which behaviours are associated with increased risk.

## Conclusions

Much work remains to be done in Sierra Leone to define the epidemiology of HIV and to develop appropriate and accessible services to address the HIV epidemic, both of which are necessary in order for the State to respect, protect, and fulfill the right to health. This study reveals a high prevalence of HIV in Kenema District among those specifically seeking HIV testing. The analysis suggests various ways in which this VCT service can be improved, including by ensuring accessibility and acceptability of the service for children and for men. The study also identifies the need for further research on the epidemiology of HIV in this region, in order for Sierra Leone to realize the right to health.

## Competing interests

The authors declare that they have no competing interests.

## Authors' contributions

FGK conceived of the study, conducted the data analysis, and drafted the manuscript. ALS, BK, and SHK were involved in the acquisition of data and contributed to revision of the final manuscript. All authors read and approved the final manuscript.

## Authors' information

FGK is a Resident in Community Medicine and PhD Student in Epidemiology at the Dalla Lana School of Public Health, University of Toronto. ALS is the Director of Disease Prevention and Control in the Ministry of Health and Sanitation of Sierra Leone. BK is the Director of the National HIV/AIDS Secretariat, Sierra Leone. SHK is the Medical Officer in Charge of HIV/AIDS Treatment and the Lassa Fever Programme at Kenema Government Hospital.

## Pre-publication history

The pre-publication history for this paper can be accessed here:

http://www.biomedcentral.com/1472-698X/10/4/prepub
